# The Rare Case of a Probably True IgE-Mediated Allergy to Local Anaesthetics

**DOI:** 10.1155/2013/201586

**Published:** 2013-05-30

**Authors:** Christina Fellinger, Felix Wantke, Wolfgang Hemmer, Gabriele Sesztak-Greinecker, Stefan Wöhrl

**Affiliations:** Floridsdorf Allergy Centre (FAZ), Franz Jonas Platz 8/6, 1210 Vienna, Austria

## Abstract

The majority of immediate type adverse reactions to local anaesthetics seem to be non-IgE-mediated. We report a case of a 31-year-old woman, who developed conjunctivitis and conjunctival erythema immediately after intrauterine application of a local anaesthetic. Skin prick testing and intradermal testing were done with lidocaine, mepivacaine, and procaine. Intradermal testing showed positive reactions to mepivacaine (1 : 10), undiluted lidocaine, and procaine (1 : 10 and undiluted). Specific IgE could be detected against mepivacaine, but not against latex. Serum tryptase was in the normal range. In order to rule out the exceptional case of a true IgE-mediated reaction, allergy testing with local anaesthetics is still required in the workup of patients.

## 1. Introduction

Many immediate-type adverse reactions to local anaesthetics (LAs) are described worldwide although the vast majority seems to be IgE independent [1]. The pathomechanisms often remain unclear, but most of the reactions are usually attributed to vasovagal reflexes. The estimated prevalence of LA hypersensitivity is reported as somehow less than 1% of applications [2]. Patients with adverse reactions to LA suffer from clinical symptoms mimicking those of anaphylaxis such as flushing, itching, hypotension, tachycardia, nausea, vertigo, bronchospasm, or collapse. The usual diagnostic work-up consists of skin prick testing, intradermal testing, and subcutaneous provocation testing. The determination of specific IgE is mainly recommended in order to exclude differential diagnoses such as latex allergy [3]. 

## 2. Case History and Methods

We report the case of a 31-year-old woman, who had developed conjunctivitis and conjunctival erythema immediately after the intrauterine application of an unknown LAs in the process of an abortion. 

In Austria, only three LA are available without the addition of epinephrine (lidocaine, mepivacaine, and procaine). Hence, we tested with the following marketed LA: lidocaine (Xylocaine 2% vial, Astra Zeneca, Austria), mepivacaine (Mepinaest purum 1% vial, Gebro Pharma, Austria), and procaine (Novanaest purum 1% vial, Gebro Pharma, Austria) at increasing concentrations of 1 : 100, 1 : 10, and undiluted. These were followed by intradermal tests (IDTs) of 0.03 mL at 1 : 10 concentration and undiluted LA. Specific IgE against lidocaine, mepivacaine, tetracaine, and articaine was measured using a classical RAST assay (Label CP Diag sprl, Nil-St-Vincent, Belgium). Specific IgE against latex and chlorhexidine was assessed by ImmunoCAP (Thermo Fisher Scientific, Upsala, Sweden). In addition, serum tryptase was determined with ImmunoCAP to rule out an underlying mast cell disease. 

## 3. Results

**Figure 1:**
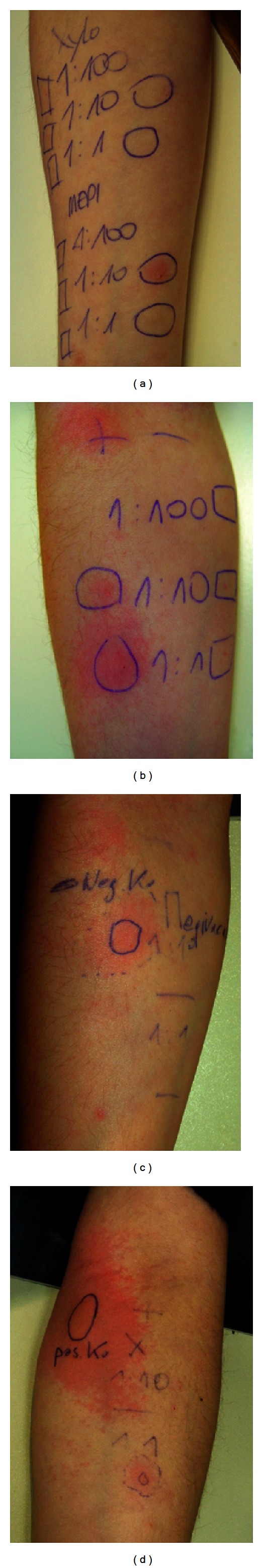
(a) Testing with xylocaine and mepivacaine showing a positive reaction of mepivacaine at 1 : 10 concentration. Undiluted LAs (marked with 1 : 1 on the skin) were not tested. (b) Positive IDT of procaine at 1 : 10 concentration and with the undiluted form. (c) Right forearm: positive IDT of mepivacaine at 1 : 10 concentration at another visit. (d) Left forearm: positive IDT of xylocaine with the undiluted form.

Astonishingly, the patient developed wheals to IDTs of the diluted amide-type LA mepivacaine (at a 1 : 10 concentration) and the undiluted lidocaine (Figure 1, Table 1). The results could be reproduced at a second occasion. As a consequence of these positive IDTs, we waived subcutaneous provocations. Then, the patient also reacted to the possible alternative ester-type LA procaine in IDTs at 1 : 10 dilution as well as in the undiluted form.

A possible IgE-mediated mechanism was further supported by an elevated signal in the nonstandardized RAST to mepivacaine (332.3 counts per minute; background: human serum albumin: 221.5 counts per minute). The summary of the results is reported in Table 2. 

Total serum IgE was normal, and the latex ImmunoCAP remained negative. Serum tryptase was within the normal range excluding mast cell activation syndrome and mastocytosis.

## 4. Discussion

Herein, we report the rare case of a possible true IgE-mediated type 1 reaction to LAs. The patient had positive IDTs to two different LAs of the amide type and one of an ester type, a reaction that was reproducible at another control visit. In the case of lidocaine, we could only detect a positive reaction with the undiluted solution which, however, can reportedly induce false positive reactions [1].

There were some recent publications about LA hypersensitivity. Bhole et al. pointed out the importance of other allergic elicitors such as chlorhexidine and latex [4]. In a Norwegian study about the work-up of 135 patients with suspected LA hypersensitivity reactions, only two patients were diagnosed as suffering from true LA allergy [5]. The first case was a delayed hypersensitivity reaction, and the second one was of the immediate type and was based on an open subcutaneous challenge test. Ten out of 135 patients were diagnosed as suffering from other IgE-mediated allergies (5/10 against chlorhexidine, 3/10 against latex, and 1/10 against triamcinolone, 1/10 against hexaminolevulinate). This was a replication of the results of the classical German study from 1997 that described allergies only in three out of 197 investigated cases (2 immediate, 1 delayed type reactions) [6]. In our own study from 2006, we could only confirm 2/36 cases [7]. In contrast, type IV allergy to LA is a relative common finding, and therefore, LAs are included in standard patch test series [8]. 

Taking together, we describe the rare case of a possible IgE-mediated reaction to an amide-type LA with cross-reactivity to an ester-type LA. Despite the dominance of non-IgE-mediated mechanisms and the less frequent non-LA type 1 allergens eliciting hypersensitivity reactions to LA, the existence of true IgE-mediated reactions cannot be completely ruled out at first hand. Hence, we think that allergy testing with LA is still required in the work-up of these patients.

## Figures and Tables

**Table 1:** Results of the intradermal provocation tests.

	Test 1		Test 2		Test 3	
Dilution	1 : 10	Undiluted	1 : 10	Undiluted	1 : 10	Undiluted
Lidocaine	neg	nd	neg	pos	nd	nd
Mepivacaine	pos	nd	pos	nd	nd	nd
Procaine	nd	nd	nd	nd	pos	pos

Histamine	nd		pos		nd	
NaCl	nd		neg		nd	

nd: not done.

**Table 2:** Results of the skin prick tests and determination of specific IgE. Specific IgE to LA was determined with a classical RAST assay (for details refer to Methods); all other in vitro tests were performed with the UniCAP system.

	Skin prick testing	Specific IgE
Lidocaine	neg	neg
Mepivacaine	neg	pos
Procaine	neg	nd

Latex	neg	<0.35 kU/L
Chlorhexidine	nd	<0.35 kU/L
Tryptase		3.2 ng/mL
Total IgE		45.8 kU/L

Histamine	pos	
NaCl	neg	
